# Supplementary material for: Trypanosomes of the Trypanosoma theileri Group: Phylogeny and New Potential Vectors
Source: Microorganisms. 2022 Jan 26;10(2):294. doi: 10.3390/microorganisms10020294 (PMC8880487; doi:10.3390/microorganisms10020294)
Supplement: Supplementary file 1 [file microorganisms-10-00294-s001.zip › Table S1. Results of deer keds screening for trypanosomes.pdf]

Table S1. Results of deer keds screening for trypanosomes. LC – Lipoptena cervi, LF – L. fortisetosa, L. sp. – Lipoptena sp., a/b/c: a–positive pools for T. theileri, b–tested pools, c–number of tested specimens. Trypanosome positive findings have bold numbers.

Site/Species	Fallow Deer			Roe Deer		Red Deer			Total
	LC	*LF*	*L.* **sp.**	*LC*	*LF*	*LC*	*LF*	*L.* **sp.**	
Blíževedly	-	-	-	0/4/4	0/4/5	-	-	-	0/4/5
Boršov nad Vltavou	0/2/2	0/1/5	-	-	0/5/25	-	-	-	0/12/36
**Bystřice**	-	-	-	0/4/4	**1/24/110**	-	-	-	**1/29/114**
Hvězda	-	-	-	-	0/3/3	-	-	-	0/3/3
Litice	0/2/2	0/4/4	-	-	-	-	-	-	0/6/6
**Mikulov**	-	0/1/1	-	-	-	-	**2/12/12**	0/1/1	**2/14/14**
Neveklov	-	-	-	0/1/3	0/7/23	-	-	-	0/8/26
Nové Strašecí	-	-	-	-	-	0/1/2	-	-	0/1/2
Obecnice	0/3/3	0/2/2	0/1/1	-	-	-	-	-	0/6/6
Planá	-	-	-	-	0/6/27	-	-	-	0/6/27
Skalka	-	-	-	0/1/1	0/3/7	-	-	-	0/4/8
Vonoklasy	-	-	-	0/1/1	-	-	-	-	0/1/1
**Total**	0/7/7	0/8/12	0/1/1	0/11/13	**1/53/200**	0/1/2	**2/12/12**	0/1/1	**3/94/248**
