# Supplementary material for: Trypanosomes of the Trypanosoma theileri Group: Phylogeny and New Potential Vectors
Source: Microorganisms. 2022 Jan 26;10(2):294. doi: 10.3390/microorganisms10020294 (PMC8880487; doi:10.3390/microorganisms10020294)
Supplement: Supplementary file 1 [file microorganisms-10-00294-s001.zip › Table S2. Results of sheep keds screening for trypanosomes.pdf]

Table S2. Results of sheep keds screening for trypanosomes. A positive locality has bold numbers. a/b: a–positive pools for *T. theileri*, b–number of tested specimens.

**Site**	**Total**
Vlkov	**3/4**
Ratíškovice	0/2
**Hořice**	**53/79**
Statenice	0/85
Přerov Předmostí	0/13
Valašská Senice	0/1
**Total**	**2/6 (33,3%)**
