# Supplementary material for: Trypanosomes of the Trypanosoma theileri Group: Phylogeny and New Potential Vectors
Source: Microorganisms. 2022 Jan 26;10(2):294. doi: 10.3390/microorganisms10020294 (PMC8880487; doi:10.3390/microorganisms10020294)
Supplement: Supplementary file 1 [file microorganisms-10-00294-s001.zip › Table S3. Infection rates of Culex mosquitoes kept in different temperature after feeding.pdf]

Table S3. Infection rates of *Culex* mosquitoes kept in different temperature after feeding.

Strain	Mosquito Species	Temperature (°C)	Total of Positive/Total of Dissected Mosquitoes
CUL59 (ex *Cs. annulata*)	*Cx. quinquefasciatus*	15	**2/15**
		21	0/33
CUL46 (ex *Cs. annulata*)	*Cx. quinquefasciatus*	8-11	**10/52**
		8-11→15	**1/14**
		15	0/54
		21	0/30
	Cx. molestus	8-11	**6/29**
		8-11→15	0/6
		15	**3/18**
		21	0/32
